# A Retrospective Analysis of 1311 Oral Surgery Procedures Performed in a Pediatric Hospital in Barcelona: A Study of Their Characteristics and Age-Related Diagnoses

**DOI:** 10.3390/jcm13185427

**Published:** 2024-09-13

**Authors:** Elvira Ferrés-Amat, Francisco Guinot-Jimeno, Ana Veloso-Durán, Josselyn Ñaupari-Pocomucha, Eduard Ferrés-Amat, Jordi Prats-Armengol, Javier Mareque-Bueno, Eduard Ferrés-Padró

**Affiliations:** 1 Service of Oral and Maxillofacial Surgery, Hospital HM Nens, HM Hospitales, 08009 Barcelona, Spain; 2 Instituto de Investigación Sanitaria HM Hospitales, Madrid, Spain; 3 Pediatric Dentistry Department, Faculty of Dentistry, Universitat Internacional de Catalunya (UIC), 08195 Barcelona, Spain; 4 Oral and Maxillofacial Surgery Service Institut Ferrés Amat, 08021 Barcelona, Spain; 5 Service of Pediatric Dentistry, Hospital HM Nens, HM Hospitales, 08009 Barcelona, Spain; 6 Oral and Maxillofacial Medicine and Oral Public Health Department, Faculty of Dentistry, Universitat Internacional de Catalunya (UIC), 08195 Barcelona, Spain; 7 Oral and Maxillofacial Department, Faculty of Dentistry, Universitat Internacional de Catalunya (UIC), 08195 Barcelona, Spain

**Keywords:** oral surgery, childhood, teenagers, surgical procedures

## Abstract

**Background:** Oral surgery involves the diagnosis and surgical treatment of diseases affecting the soft and hard tissues of the oral cavity and encompasses a wide range of surgical interventions. The aim of this investigation was to study the characteristics and age-related diagnoses of these oral surgeries, as well as to describe the surgical procedures performed in a pediatric oral and maxillofacial surgery service. **Methods**: A descriptive, retrospective, observational, and relational study was conducted on children and adolescents aged from 0 to 22 years who were treated in a pediatric oral and maxillofacial surgery service at a children’s hospital. **Results:** We analyzed 1311 surgical interventions (51.4% were on boys and 48.6% on girls), consisting of 24.8% soft tissue surgeries, 65.9% bone and dental tissue surgeries, and 9.3% mixed tissue surgeries. The most common pathologies were tooth eruption disorders (65.9%), followed by ankyloglossia (20.5%). The most frequent treatment was wisdom teeth extraction (31.3%). A statistically significant association (*p* < 0.05) was found between surgical treatments and variables such as age, sex, tissue type, and biopsy. **Conclusions:** This study enhances our understanding of pediatric oral surgery, emphasizing that the most common pathology is altered tooth eruption, while the most frequent surgical intervention is the extraction of wisdom teeth at different stages of development.

## 1. Introduction

Oral surgery involves diagnosing and surgically treating diseases that can affect the soft and hard tissues of the oral cavity [1,2]. As clinicians, we are able to identify morphological and functional alterations related to oral and maxillofacial development and growth [3,4]. The potential for adverse effects on growth, behavioral orientation, and peri- and post-operative management necessitates special considerations. These include obtaining parental consent, ensuring biosafety, understanding the developing anatomy and dentition, accurately diagnosing the need for treatment, and selecting appropriate instruments and techniques. This field encompasses surgeries of medium complexity which are performed under one of the following conditions: (1) general anesthesia, (2) intravenous sedation, or (3) locoregional or local anesthesia. These procedures do not require hospital admission post-operation [5,6].

Pediatric patients may present a wide variety of intraoral soft tissue pathologies, the most common being mucocele and inflammatory lesions [7]. These lesions affect the oral and maxillofacial complex of children and adolescents and may range from benign lesions to exceptionally malignant tumors with aggressive behavior [8]. It is considered standard care to send any pathological tissue removed from the oral and maxillofacial region for histopathological examination [9].

In Spain, the Maxillofacial Surgery Services within the hospital system commonly perform a range of oral surgical procedures. These include the extraction of teeth and root fragments, removal of impacted or unerupted teeth, and the extraction of supernumerary teeth. Other procedures involve dental fenestration for orthodontic treatment, extraction of maxillary cysts, and biopsies of hard and soft tissues. Additionally, frenulum surgery, removal of orthodontic maxillary plaques and wires, and minor soft-tissue surgery are also performed. The scope of these services extends to cryosurgery for small soft-tissue injuries, as well as arthroscopy and arthrocentesis of the temporomandibular joint. Finally, the extraction of salivary gland stones is also a part of the procedural repertoire. [10,11].

Taking into account the importance of teamwork between different pediatric hospital services, is important to review the therapeutic protocols offered by pediatric dental surgery. This involves a thorough examination and updating of oral surgical protocols. This paper is intended to enhance the knowledge of oral and maxillofacial surgery procedures among pediatric dentists, oral and maxillofacial surgeons, and pediatricians. It underscores the importance of teamwork in treating pediatric patients, from newborns to teenagers or young adults. Thus, the aim of this study was to describe oral surgical interventions in children and young patients treated in the pediatric oral and maxillofacial surgery service of HM Nens, HM Hospitales, in Barcelona. Subsequently, this study aimed to compare the results obtained from these interventions with other previously published studies and analyze the relationship between patient characteristics and age-related diagnoses associated with oral surgical interventions in children and young patients. This provides relevant information and serves as a guide for accurate diagnosis and better understanding of the treatment needs in pediatric oral surgery.

## 2. Materials and Methods

### 2.1. Study Design and Population

A retrospective, descriptive observational study was conducted, reviewing all cases of patients aged 0 to 22 years treated between 2015 and 2019 by the Oral and Maxillofacial Surgery Service of Hospital HM Nens, HM Hospitales, in Barcelona. To estimate the proportion of surgeries, pathologies, and treatments with a precision of 3%, a minimum sample size of 1068 was required (considering *p* = q = 0.5).

The study population included newborns, children, teenagers, and young adults who were treated according to pediatric hospital protocols, giving complete care during the growth and development of the oral cavity and maxillofacial area. Some patients begin orthodontic treatment in their late teens, end later than 18 years, and require oral and maxillofacial surgery between the ages of 18 and 22.

### 2.2. Ethical Approval

This study was approved by the Scientific Council and the Ethics Committee (CEIm) of the HM Hospitales. The approval certification was obtained on 19 October 2022 (Approval Reference: Minute No. 260, CEIm HM Hospitales code: 22.10.2080GHM).

### 2.3. Patients and Inclusion/Exclusion Criteria

The population studied consisted of newborns, children, teenagers, and young adults with the following inclusion criteria: healthy patients without any diagnosis of disease syndrome who were eligible for surgical intervention, cases attended in our service in the period 2015–2019, aged between 0 and 22 years. The exclusion criteria were patients with a syndrome or disease and those over 23 years of age.

All patients were diagnosed and treated by the Oral and Maxillofacial Surgery team, which consisted of five experts who were previously trained and calibrated examiners using the same diagnostic criteria. Inter-examiner reliability was assessed by analyzing 20 cases not included in the study, which were re-evaluated after one week, resulting in an inter-examiner Kappa index. All interventions were carried out in the surgical area. The surgical programs’ lists from the Service of Oral and Maxillofacial Surgery were utilized, and the medical histories of patients who underwent surgery within the study period were reviewed from these lists.

Depending on the pathology, additional radiographic exams (panoramic radiography, intraoral-dental radiography, computerized tomography (CT), cone bean computerized tomography (CBCT), bone gammagraphy, or magnetic resonance imaging (MRI)) were required for correct diagnosis and to be able to individualize the treatment of each patient.

### 2.4. Data Confidentiality and Statistical Analysis

The information was treated confidentially from the electronic database of surgical schedules. The following variables were evaluated: gender, age, date of surgery, place where the intervention was performed, type of anesthesia, classification of soft tissue, hard or mixed tissue, pathology, treatment, biopsy performed based on individual case requirements, and complications.

A coded template was used to ensure patient anonymity, guaranteeing the confidentiality of personal data. Data were registered in a database, then correlated in a Microsoft Office Excel sheet and analyzed statistically. Qualitative variables were described in terms of frequency and percentage, while quantitative variables were summarized with mean, median, standard deviation, quartiles, and range. The Kruskal–Wallis test was used to compare more than two groups for quantitative variables, and the chi-square test was used to compare two groups for qualitative variables. All analyses were conducted using R Version 4.1.1 software (R Foundation for Statistical Computing. Crore Team, Vienna, Austria), with a *p*-value of ≤0.05 and a 95% confidence level considered to be statistically significant.

## 3. Results

### 3.1. General Descriptive Analysis

A total of 1311 patients who underwent surgery between January 2015 and December 2019 were reviewed. Of these patients, 51.4% were male and 48.6% were female. The age of the patients ranged from 0 to 22 years with a mean age of 12.91 ± 5.79. Our work center is a pediatric hospital where diagnoses and treatments are performed from birth, childhood, adolescence, and young adulthood. For this reason, our study includes treatments for patients aged 0 to 22 years old in order to provide comprehensive care that includes patients who begin orthodontic treatment at the end of their adolescence.

Analyzing the surgical interventions based on tissue type, pathology, type of anesthesia, and histopathological study (biopsy), the following results were observed. Regarding tissue type, the distribution was as follows: 24.8% of the interventions involved soft tissue treatments, 65.9% involved bone and dental tissue treatments, and 9.3% involved mixed tissue treatments. In terms of pathology, the distribution was as follows: 65.9% for eruption-related issues, 20.5% for tongue tie, 8.3% for osteomyelitis/osteitis, and 4.3% for soft-tissue lesions such as mucocele and salivary gland issues. Concerning anesthesia techniques, 96.6% of the surgical treatments were performed using local anesthesia combined with intravenous sedation, while 3.4% required general anesthesia. Finally, histological samples for study were obtained in 64.4% of the surgeries.

Studying the prevalence of oral and maxillofacial surgery treatments, the results establish that the most frequent treatments were the extractions of included wisdom teeth (31.3%) and supernumerary teeth (18.8%). Other notable surgical treatments included lingual frenectomies (13.6%) and retained tooth fenestrations (8.4%). Additionally, fractures accounted for 0.4% of cases, orthognathic surgeries for 0.6%, and labial frenectomies for 0.9%.

### 3.2. Surgical Procedures and Age

We studied the relationship between oral and maxillofacial surgery treatments in childhood and age (Table 1). The analysis showed that the extraction of impacted wisdom teeth was typically performed at a mean age of 18.25 years (±2.64), with a median age of 19 years. Fenestration of retained teeth occurred at a mean age of 14.85 years (±2.15), with a median age of 15 years. Dental extractions were conducted at a mean age of 12.12 years (±3.85), with a median age of 12 years. Mucocele excision was carried out at a mean age of 11.08 years (±3.17), with a median age of 11 years. The extraction of supernumerary teeth had a mean age of 10.34 years (±3.14), with a median age of 10 years. Ankyloglossia treatments, including lingual frenotomy or frenectomy, were carried out at a mean age of 8.72 years (±3.52), with a median age of 9 years. Lastly, dental implants were placed at a mean age of 19.53 years (±1.81), with a median age of 20 years.

In terms of age-specific treatment patterns, frenotomy was the most frequent treatment in the 0–3 years age group, accounting for 75.2% of cases. For the 4–7 years age group, the predominant treatments were lingual frenectomy and plasty, comprising 38.4% of cases. In the 8–10 years age group, surgical extractions were most common, making up 33.3% of the treatments. For the 12–15 years, 16–19 years, and 20–22 years age groups, the most frequent treatment was the extraction of wisdom teeth, with frequencies of 59.5%, 86%, and 61.5%, respectively (Table 2). Statistical analysis revealed significant differences between surgical treatments and age (*p* < 0.001), indicating a strong association between the type of surgical intervention and the patient’s age.

### 3.3. Surgical Procedures and Gender

**Figure 1:**
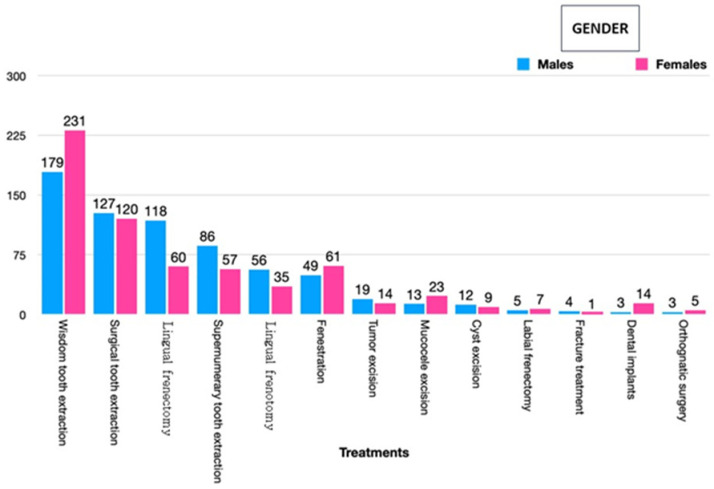
Association between oral and maxillofacial surgery treatments and gender in childhood and adolescence in a pediatric hospital.

The results regarding the association between oral and maxillofacial surgery treatments and gender are detailed in Table 3 and Figure 1. Among males, the distribution of treatments was as follows: 26.6% for extractions of impacted wisdom teeth, 18.8% for general extractions, 17.5% for lingual frenectomy and plasty, 12.8% for extractions of supernumerary teeth, 8.3% for frenotomy, and 7.3% for fenestrations. In contrast, the distribution for females was different: 36.6% for extractions of impacted wisdom teeth, 9.6% for retained tooth fenestrations, 9.4% for lingual frenectomy, 8.9% for extractions of supernumerary teeth, and 5.5% for lingual frenotomy. Statistical analysis revealed significant differences (*p* < 0.001) in the frequencies of impacted wisdom tooth extractions and lingual frenectomy treatments between genders. Specifically, females exhibited a higher percentage of wisdom tooth extractions and dental implants, whereas males had a higher percentage of lingual frenectomies and extractions of supernumerary teeth.

### 3.4. Histopathologic Study and Surgical Procedure

Table 4 presents the results of the association between oral and maxillofacial surgery treatments in childhood and the use of biopsies based on individual case requirements. Treatments that were biopsied 100% of the time included mucocele excision, tumor excision, and cyst excision. Conversely, frenotomy, labial frenectomy, orthognathic surgery, dental implants, and fractures were never biopsied. Among other treatments, the extractions of impacted wisdom teeth had a biopsy rate of 93.9%, with 6.1% not biopsied. For extractions of supernumerary teeth, 86.7% were biopsied and 13.3% were not. Surgical dental extractions had a biopsy rate of 80.2%, with 19.8% not biopsied. Fenestrations of teeth showed a biopsy rate of 72.7%, with 27.3% not biopsied. The surgical treatments that were not biopsied was due to the fact that, during the surgery, no sufficient pathological tissue sample (neither soft nor bone) was obtained for histopathological study. A statistical analysis revealed significant differences (*p* < 0.001) in the use of biopsies across most surgical treatments, except for fenestration treatments, which showed a non-significant *p*-value of 0.061.

## 4. Discussion

This study aimed to describe oral and maxillofacial surgical interventions and their distribution based on age, gender, pathology, and type of surgery in pediatric patients at a specialized pediatric hospital. The hospital features a comprehensive pediatric dentistry department offering a range of services, including conservative dentistry, orthodontics, dentofacial orthopedics, speech and myofunctional therapy, pediatric and adolescent dentistry, endodontics, prosthodontics, esthetic rehabilitation, oral hygiene, and prophylaxis. Patients from birth through adolescence and young adulthood received care from the oral and maxillofacial surgery service. Over a period of five years, a total of 1311 surgical interventions were analyzed, distinguishing this study from others that include both children and adults.

### 4.1. Age and Gender

In our study, the age of the patients ranged from 0 to 22 years, with a mean age of 12.91 ± 5.79 years, encompassing individuals from birth through adolescence and young adulthood. To contextualize our findings, we compared our results with those from four key studies. Leco-Berrocal et al. [10] included a diverse age range from 21 to 40 years, with additional groups under 20 years, 41–64 years, and over 65 years. Capurro et al. [1] focused on children aged 0–14 years, while Jokić et al. [12] had a population with an average age of 38.7 ± 19.4 years. Pérez-Garcia et al. [5] studied children under 18 years. Regarding gender distribution, our study observed a higher incidence in males (51.4%) compared to females (48.6%), which is consistent with Capurro et al. [1], where 51.8% of the patients were male and 48.2% were female.

### 4.2. Pathology

In our study, the most frequent pathology was dental eruption anomalies, which accounted for 65.9% of cases and involved various surgical treatments. This prevalence is notably higher compared to the 36.7% reported by Patil et al. [13] in their study of 1519 patients. The early diagnosis of dental eruption issues is crucial for effective treatment planning. At our hospital, dental check-ups include radiological examinations such as orthopantomography, which aids in the early detection of pathologies requiring surgical intervention. This proactive approach may contribute to the higher prevalence of dental eruption pathology observed in our study compared to other studies.

### 4.3. Surgical Procedure

Surgical treatments were performed under local anesthesia and sedation with a percentage greater than 96.6% and general anesthesia with a percentage of 3.4%.

Following the work protocol, we performed biopsies in the treatments of wisdom tooth, supernumerary teeth, and fenestrations, provided that, during the surgical act, soft tissue was obtained (dental follicle); if we did not find soft tissue sample, no histopathological study was performed.

#### 4.3.1. Surgical Extraction

##### Surgical Extraction Wisdom Tooth

In our study, the most common surgical intervention was the extraction of impacted third molars, performed in 65.9% of cases involving dentoalveolar tissues. This procedure was most prevalent in patients aged 16–20 years, accounting for 31.3%. In comparison, Leco-Berrocal et al. [10] reported a higher prevalence of 83.5%, while Pérez-Garcia et al. [5] found a prevalence of 55.6%. Chaparro-Avendaño et al. [14] reported a significantly lower prevalence of 8.64%. Our findings also revealed that the impaction of third molars is more common in females (36.3%) compared to males. This pattern may be related to the earlier eruption of third molars in females, which aligns with general growth patterns in the oral and maxillofacial region. The treatment of wisdom teeth in the 8–11 year age group is related to the pathology of permanent second molar eruption. Our work center follows the guidelines recommended in the protocol [9], performing preventive extraction of the wisdom tooth to allow and favor the development and eruption of the second permanent molars.

##### Surgical Dental Extraction

The prevalence and reasons for surgical extractions offer insights into dental treatment needs, with caries being the most frequent cause, followed by impacted teeth. In our study, the prevalence of surgical extraction was 18.8%, with an age range that oscillated between 8 and 16 years; there was no significant difference in sex. In contrast, Ashiwaju et al. [15] reported a higher prevalence of 58.8% in a population of 235 children, with an age range from 3 to 15 years and a notable predominance of male patients undergoing extractions. Additionally, Jokić et al. [12] found that dental extractions were the most common procedure, accounting for 37.67% of cases among 2201 out of 11,680 patients treated under local anesthesia in an outpatient setting. Their study also highlighted a greater prevalence of dental extractions in females, with an average age of 38.7 ± 19.4 years.

Lim et al. [16] reported on the management of odontogenic infections in pediatric patients (under 18 years), noting that 83.7% were managed as outpatients, while 7% underwent extractions in a surgical setting. Inpatient admissions averaged 3 days, and 68% of patients received definitive treatment through dental extraction, which was performed under local or general anesthesia. Pérez-Garcia et al. [5] analyzed 3187 oral surgeries, finding a notable prevalence of 89.2% for dental extractions. Of these, 487 extractions were performed on patients under 18 years, primarily targeting the first permanent molars extracted due to caries, which made up 36% of the extractions. In contrast, our study categorized extractions of first permanent molars under the pathology of osteomyelitis/osteitis, representing 8.3% of cases. Capurro et al. [1] examined 1667 patients treated on an outpatient basis and 832 in the operating room. They found that 20.88% of extractions were performed on patients aged 8 to 11 years, with a slightly higher prevalence in males (51.8%). Their study used local anesthesia and sedation in 4.8% of the cases, general anesthesia in 47.12% of the cases, with most of the surgeries conducted in the operating room, differing from our study’s approach.

##### Supernumerary Teeth Extraction

In our study, the prevalence of supernumerary tooth extractions was 10.9%, which is lower than the 25.5% reported by Pérez-Garcia et al. [5]. Our study included patients aged 8–14 years and observed a higher incidence in males (12.8%), consistent with the findings of Ferrés-Padró et al. [3] and Fernández-Montenegro et al. [17]. Ferrés-Padró et al. [3] studied patients aged 5–19 years, while Fernández-Montenegro et al. [17] covered ages 5–56 years. Both studies similarly noted a higher prevalence of supernumerary extractions in males.

#### 4.3.2. Tooth Fenestration

The growing demand for orthodontic treatments has led to an increase in surgical interventions involving canines, including fenestrations. In our study, fenestrations for retained teeth accounted for 8.4% of cases, primarily involving patients aged 12–17 years and a prevalence of 9.6% in females. In contrast, Pérez-Garcia et al. [5] reported only one case of fenestration, indicating a lower prevalence compared to our findings. In our pediatric hospital, we have an orthodontic service that refers patients requiring orthodontic-surgical treatment such as fenestrations. These treatments are typically indicated during the final mixed dentition or definitive dentition stages, which align with the patient’s chronological age. Our results reflect this practice, demonstrating that fenestrations are performed in accordance with the recommended timing for such interventions.

#### 4.3.3. Dental Implants

The use of implants in younger patients has been a subject of debate. However, using the appropriate preventive measures, the clinicians can enhance quality of life, esthetics, and functionality for children and adolescents until their growth is complete, at which point more extensive rehabilitative treatments can be considered [18]. In our study, dental implants were among the less-frequently performed treatments, accounting for only 1.3% of cases, with patients aged 17–21 years, and a higher incidence observed in females (2.2%). The use of mini-implants as temporary solutions for provisional prosthetic rehabilitation in growing patients presents a promising alternative [19,20].

#### 4.3.4. Orthognathic Surgery

The ideal timing for orthognathic surgery in growing patients remains controversial, primarily due to the potential impact of the procedure on continued facial growth [21]. In our study, orthognathic surgery was one of the least-frequent treatments, with a prevalence of 0.6% among patients aged 12–22 years and a slightly higher incidence in females (0.8%). This procedure was classified within the pathology of dentofacial deformities (0.6%). Orthognathic surgery can be performed in conjunction with various treatment modalities to correct facial deformities of diverse etiologies [22].

#### 4.3.5. Lingual Frenotomy and Frenectomy and Plasty

In our study, ankyloglossia was the second most common pathology, accounting for 20.5% of cases. This prevalence is higher than the 4.2% to 10.7% range reported for newborns by Rowan-Legg [23] and the 15.5% prevalence observed in another study [24]. Ferrés-Amat et al. [25] reported a notably higher prevalence of ankyloglossia, with 64.9% of cases occurring in males. The preferred treatment for ankyloglossia in childhood is frenectomy and plasty [25]. In our study, frenotomies were performed in 6.9% of cases, with a higher prevalence in males (8.3%). Among soft-tissue surgeries, lingual frenectomy was the most common, comprising 13.6% of the procedures, with patients aged 3–15 years. The overall prevalence of frenectomies in our study was 14.49%, including 178 lingual and 12 labial frenectomies. Consistent with our findings, Chaparro-Avendaño et al. [14] also reported a lower prevalence of labial frenectomy (0.9%) compared to lingual frenectomy. In our study, lingual frenectomies were more prevalent among males (17.5%), while labial frenectomies were more common in females (1.1%).

#### 4.3.6. Fractures of the Maxillary Bones and Dental Trauma

More than 25–30% of children suffer orofacial trauma; however, maxilla and mandible fractures are very uncommon [26]. In contrast, dental or dentoalveolar fractures are quite common in early childhood and are frequently managed in the emergency room of the Paediatric Dentistry Service [27]. In our study, maxillofacial fractures were the least-frequent treatment, with a prevalence of 0.4%. These fractures were most commonly observed in males (0.6%) and primarily affected patients aged 10–17 years. This is consistent with previous studies: Segura-Palleres et al. [28] reported that in a multicentric sample of 322 patients (aged 0-18 years), with maxillofacial trauma lesions were treated, with a mean age of 14 years; similarly, Cleveland et al. [29], studying 5568 patients aged 0–18 years, found a mean age of 12 years and a male prevalence of 68.3%. Both studies [28,29] noted an increase in incidence with age and a male predominance, aligning with the findings of our study.

### 4.4. Soft Tissue Pathology

The distribution according to tissue type was 24.8% of soft-tissue treatments. Mucocele is the most common lesion of the oral mucosa in children and adolescents, and was one of the treatments included in this pathology. In our study, the prevalence of mucocele exeresis was 27.7% with an age of 8–14 years, highlighting a 3.6% prevalence in females. This is in contrast to Pérez-Garcia et al. [5], who reported a much lower prevalence of mucocele excision at only 0.6%, with cases occurring in patients aged 15–17 years. Their study identified three cases of soft-tissue pathology, with histopathological diagnoses confirming two mucoceles and one dermoid cyst [5].

Wu et al. [7] studied 64 patients under 18 years with histopathological-confirmed mucoceles, comprising 34 girls and 30 boys. Additionally, Miranda et al. [30] reported that mucoceles accounted for approximately 3.6% to 6.1% of all lesions diagnosed, with a higher prevalence in females (56.2%).

### 4.5. Procedures on Soft Tissue and Bone (Mixed)

In our study, treatments involving mixed tissues, specifically fenestration and labial frenectomy, constituted 9.3% of cases. All soft-tissue pathologies underwent biopsy, whereas for bone-dental tissues, biopsy was performed based on individual case requirements, with no complications reported for any treatments.

A significant limitation of our study is the scarcity of published research focused on pediatric oral surgery. Most existing studies either address surgical treatments in adult populations or include both adults and children. Our study specifically examines pediatric patients—children and adolescents— and young adults treated in a specialized pediatric oral and maxillofacial surgery service within a dedicated pediatric hospital.

## 5. Conclusions

This study aimed to raise the understanding of oral and maxillofacial surgery procedures among pediatric dentists, oral and maxillofacial surgeons, and pediatricians. We can observe the importance of the different specialist’s teamwork in the treatment of the pediatric patient, from the newborn to the teenager and young adulthood.

Our findings indicate that the most frequent treatment across the study was the extraction of impacted wisdom teeth. Among the youngest patients (0–3 years), the predominant treatment was lingual frenotomy, while lingual frenectomy and plasty were most common in the 4–7 years age group. For the group of 8–11 years, it was surgical dental extractions. In older age groups (12–15, 16–19, and 20–22 years), wisdom tooth extraction emerged as the most commonly performed procedure.

Furthermore, we observed that extractions of impacted wisdom teeth and dental implants were more prevalent in females, whereas treatments for ankyloglossia (frenotomy or frenectomy) and extractions of supernumerary teeth were more frequent in males.

## Figures and Tables

**Table 1:** Distribution of oral and maxillofacial surgery treatments in childhood and adolescence based on the age of the patient when treated at a pediatric hospital.

			Percentile		
TREATMENT	MEDIA (DS)	Min–Max	25	50	75
Wisdom teeth extractions	18.06 (±2.31)	8–22	17.00	18.00	20.00
Surgical tooth extraction	12.12 (±3.85)	2–22	9.00	12.00	14.00
Ankyloglossia treatment	8.72 (±3.52)	0–18	7.00	9.00	11.00
Supernumerary tooth extraction	10.34 (±3.14)	4–22	8.00	10.00	12.00
Retained teeth fenestration	14.85 (±2.15)	8–19	13.00	15.00	16.00
Mucocele excision	11.08 (±3.17)	6–16	9.00	11.00	14.00
Tumor excision	13.00 (±4.19)	6–22	11.00	13.00	15.00
Cyst excision	13.81 (±3.27)	7–20	12.00	14.00	15.00
Dental implants	19.53 (±1.81)	15–22	19.00	20.00	20.00
Labial frenectomy	11.33 (±4.60)	2–15	9.50	14.00	14.50
Orthognathic surgery	17.38 (±6.04)	9–22	13.00	19.00	22.00
Fracture treatment	12.80 (±3.63)	10–19	11.00	11.00	13.00
Kruskal–Wallis (*p* ≤ 0.05)					

**Table 2:** Distribution of treatments by age group.

AGE						
	0–3 Years	4–7 Years	8–11 Years	12–15 Years	16–19 Years	20–22 Years
TREATMENT						
Wisdom teeth extractions	0 (0.0%)	0 (0.0%)	21 (5.7%)	262 (59.5%)	117 (86.0%)	8 (61.5%)
Surgical tooth Extraction	4 (3.3%)	62 (26.7%)	123 (33.3%)	51 (11.6%)	5 (3.7%)	2 (15.4%)
Lingual frenectomy	22 (18.2%)	89 (38.4%)	58 (15.7%)	9 (2.0%)	0 (0.0%)	0 (0.0%)
Supernumeraries Teeth extractions	0 (0.0%)	57 (24.6%)	69 (18.7%)	14 (3.2%)	0 (0.0%)	1 (7.7%)
Retained Teeth Fenestration	0 (0.0%)	1 (0.4%)	39 (10.6%)	70 (15.9%)	0 (0.0%)	0 (0.0%)
Lingual frenotomy	91 (75.2%)	0 (0.0%)	0 (0.0%)	0 (0.0%)	0 (0.0%)	0 (0.0%)
Mucocele excision	0 (0.0%)	12 (5.2%)	18 (4.9%)	6 (1.4%)	0 (0.0%)	0 (0.0%)
Tumor excision	0 (0.0%)	0 (0.0%)	18 (4.9%)	9 (2.0%)	0 (0.0%)	1 (7.7%)
Cyst excision	0 (0.0%)	0 (0.0%)	11 (3.0%)	7 (1.6%)	1 (0.7%)	0 (0.0%)
Dental implants	0 (0.0%)	0 (0.0%)	0 (0.0%)	0 (0.0%)	10 (7.4%)	7 (1.6%)
Labial frenectomy	2 (1.7%)	1 (0.4%)	6 (1.6%)	3 (0.7%)	0 (0.0%)	0 (0.0%)
Orthognathic surgery	0 (0.0%)	0 (0.0%)	0 (0.0%)	1 (0.2%)	3 (2.2%)	0 (0.0%)
Fracture treatment	0 (0.0%)	0 (0.0%)	4 (1.1%)	1 (0.2%)	0 (0.0%)	0 (0.0%)

**Table 3:** Association of oral and maxillofacial surgery treatments in childhood and adolescence performed in a pediatric hospital, according to gender. * Statistical analysis revealed significant differences (*p* ≤ 0.05).

	GENDER				
	Male		Female		
TREATMENT	N	%	N	%	*p*
Wisdom tooth extractions	179	26.6%	231	36.3%	<0.001 *
Surgical tooth extraction	127	18.8%	120	18.8%	0.998
Lingual frenectomy	118	17.5%	60	9.4%	<0.001 *
Supernumerary tooth extraction	86	12.8%	57	8.9%	0.033 *
Lingual frenotomy	56	8.3%	35	5.5%	0.050 *
Fenestration	49	7.3%	61	9.6%	0.132
Mucocele excision	13	1.9%	23	3.6%	0.065
Tumor excision	19	2.8%	14	2.2%	0.487
Cyst excision	12	1.8%	9	1.4%	0.664
Dental implants	3	0.4%	14	2.2%	0.006 *
Labial frenectomy	5	0.7%	7	1.1%	0.570
Orthognathic surgery	3	0.4%	5	0.8%	0.495
Fracture treatment	4	0.6%	1	0.2%	0.375
Pearson’s chi-square (*p* ≤ 0.05)					

**Table 4:** Association of oral and maxillofacial surgery treatments in childhood and adolescence performed in a pediatric hospital, and biopsy. * Statistical analysis revealed significant differences. Fisher’s exact test (*p* ≤ 0.05). Pearson’s Chi-square (*p* ≤ 0.05).

	BIOPSY				
	YES		NO		
TREATMENT	N	%	N	%	*p*
Wisdom tooth extractions	385	93.9%	25	6.1%	<0.001 *
Surgical tooth extraction	198	80.2%	49	19.8%	<0.001 *
Lingual frenectomy	0	0.0%	178	100.0%	<0.001 *
Supernumerary tooth extraction	124	86.7%	19	13.3%	<0.001 *
Fenestration	80	72.7%	30	27.3%	0.061
Lingual frenotomy	0	0.0%	91	100.0%	<0.001 *
Mucocele excision	36	100.0%	0	0.0%	<0.001 *
Tumor excision	33	100.0%	0	0.0%	<0.001 *
Cyst excision	21	100.0%	0	0.0%	0.011 *
Dental implants	0	0.0%	17	100.0%	<0.001 *
Labial frenectomy	0	0.0%	12	100.0%	<0.001 *
Orthognathic surgery	0	0.0%	8	100.0%	<0.001 *
Fracture treatment	0	0.0%	5	100.0%	0.006 *

## Data Availability

The data presented in this study are available on request from the corresponding author. The data are not publicly available due to ethical requirements.
